# The Negative Effect of Ability-Focused Praise on the “Praiser’s” Intrinsic Motivation: Face-to-Face Interaction

**DOI:** 10.3389/fpsyg.2020.562081

**Published:** 2020-11-24

**Authors:** Kyosuke Kakinuma, Fumika Nishiguti, Kotoe Sonoda, Haruhi Tajiri, Ayumi Tanaka

**Affiliations:** ^1^Faculty of Psychology, Doshisha University, Kyoto, Japan; ^2^Japan Society for the Promotion of Science, Tokyo, Japan

**Keywords:** motivation, praise, ability, interpersonal interaction, eye-tracking

## Abstract

Most previous research has demonstrated that receiving ability-focused praise (e.g., “You are smart”) negatively affects intrinsic motivation following failure. Surprisingly, a recent study showed that ability-focused praise affects not only the praisee but also the person offering praise, that is, the praiser. However, evidence of the effects on the praiser is quite limited, despite the utility of praise in education. Therefore, the present study employed face-to-face interaction to advance the knowledge of the effects of praise on the praiser. Two experiments were conducted in which undergraduate participants (*n* = 39 and *n* = 51) praised a research confederate. We measured attentional engagement using an eye-tracker as a behavioral indicator of intrinsic motivation, as well as self-reported task enjoyment. To estimate the effect of praise, we combined the results of two experiments and conducted a Bayes factor meta-analysis. The results showed that in the ability praise group, participants’ attentional engagement in a task was significantly lower than in the control group. The present finding indicates that ability-focused praise negatively affects the praiser’s intrinsic motivation and suggests that praise should be used with caution in social and educational contexts.

## Introduction

Praise has received considerable attention in schools. In Japan, for example, not only teachers are encouraged to praise students (Japanese Ministry of Education, Culture, Sports, Science and Technology, 2018) but also students are encouraged to praise each other (e.g., Fukuoka-Ken Board of Education, 2015). The vast majority of empirical research has examined the effects of praise and demonstrated various effects on the praisee depending on the type of praise (see Henderlong and Lepper, 2002, for a review). Surprisingly, a recent study found that praise affects not only the praisee but also the one offering the praise, that is, the praiser (Kakinuma et al., 2020). Considering the popularity of praise in schools, investigation of the understudied effects of praise on the praiser is very relevant in the school context.

Much research has revealed that the effect of receiving praise depends on the type of praise; some types of praise negatively affect the praisee’s motivation (see Henderlong and Lepper, 2002). For example, Mueller and Dweck (1998) found that receiving ability-focused praise negatively affected an individual’s intrinsic motivation following failure. People who received ability-focused praise tended to evaluate their performance in light of a limited ability or trait and, consequently, were less intrinsically motivated. Research on motivation has continued to report similar findings concerning the effects of ability-focused praise (e.g., Skipper and Douglas, 2012; Zentall and Morris, 2012; Brummelman et al., 2014; Morris and Zentall, 2014).

Recently, a study on peer praise argued that praise between students affects not only the praisee but also the praiser (Kakinuma et al., 2020). Kakinuma et al. (2020) showed that ability-focused praise negatively affects the praiser’s intrinsic motivation. In their experiment, participants read a scenario in which a junior student succeeded (Study 2) or observed another student working on a task through a monitor (Study 3). Then, they were asked to generate feedback-based sentences for the student, involving ability- or effort-focused praise or objective feedback. Then, the participants worked on a difficult task and failed. Subsequently, they were asked to rate their enjoyment of the task. The results showed that participants in the ability-focused group reported less task enjoyment than those in the control group. This finding suggests that peer praise may have the same effect on the praiser and the praisee.

Praise is defined as a subset of feedback that contains a positive evaluation (Henderlong and Lepper, 2002; Hattie and Timperley, 2007), and peer feedback also has an effect on the giver (provider) as well as the receiver (Topping, 1998; Cho and Cho, 2011; Deiglmayr, 2018). For example, a literature review by Van Popta et al. (2017) showed that “providing peer feedback can be beneficial for the provider” (p. 29); giving feedback encourages critical thinking and develops skills in making evaluative judgments. Moreover, Deiglmayr (2018) suggested that providing feedback may lead to a deeper reflection on the cognitive strategies and, in turn, improve the giver’s performance.

A plausible explanation of the effect of praise on the praiser relies on a series of findings related to the “saying is believing” effect; namely, beliefs follow from what a person says (e.g., Higgins and Rholes, 1978). Communication research has found that communicating a message about a target person modified the communicator’s recall of the target person’s behavior (Higgins and Rholes, 1978; Todorov, 2002). Higgins and Rholes (1978) explained that a communicator would assign a label to a target person through communicating the message, and that the label would become part of the available information associated with the target. Based on these studies, the ability-focused praiser would assign an ability-focused label to the performance, which would lead the praiser to evaluate his or her performance on the ability by connecting the representation of performance to the evaluation of ability (Kakinuma et al., 2020). That would decrease the praiser’s intrinsic motivation, similar to the effects on the praisee.

Given the utility of praise in schools and the relevance of praise for students, it is of practical importance to examine the effects of praise on the praiser. However, the evidence is quite limited. First, Kakinuma et al.’s (2020) study did not involve face-to-face interaction; thus, it is hard to know whether it captures a real-life phenomenon or how applicable it is to social and educational contexts. Considering that praise is social interaction, and offering praise can include diverse communicational or educational purposes (Kanouse et al., 1981; Henderlong and Lepper, 2002), further work should be conducted to verify the effects on the praiser in face-to-face settings. Second, the direct effect was found on intrinsic motivation using only a self-reported scale. Some motivational research emphasizes behavioral measures of intrinsic motivation because they are thought to be more ecologically valid than self-reported scales (Corpus and Lepper, 2007; Wiechman and Gurland, 2009). Thus, the effect should be validated using behavioral measures of intrinsic motivation.

For these reasons, the present study aimed to advance the knowledge of the effect of praise on the praiser through face-to-face interaction. We measured intrinsic motivation for the task using a behavioral measure as well as a self-reported scale. Considering the recent prevalence of peer feedback in education, we focused on praise between colleagues (Van Gennip et al., 2009; Van Popta et al., 2017). The present study compared ability-focused praise with feedback that does not include praise, although previous research has examined the effects of effort-focused praise as well as ability-focused praise (e.g., Mueller and Dweck, 1998). Recent studies on praisees and praisers have suggested that the effects of effort-focused praise were complicated and related to certain moderators (e.g., Amemiya and Wang, 2018; Kakinuma et al., 2020). As investigating moderators falls beyond the scope of this study, we focused on ability-focused praise. Based on the assumption that praise would have the same effect on the praiser as on the praisee (Kakinuma et al., 2020), ability-focused praise would negatively affect the intrinsic motivation for the task measured by the self-report and behavioral indexes.

Using a face-to-face setting, we set a task-related exchange between students. In the task-related exchange, age-matched students worked on a task, a student provided his or her colleague with performance feedback, and then the student offered ability-focused praise to him or her. We used various types of performance feedback phrases to cover a wider educational setting. Some studies used feedback comments (e.g., “You did well”) (e.g., Mueller and Dweck, 1998; Kamins and Dweck, 1999), whereas others used objective feedback (“You got 5 out of 5 correct”) (e.g., Skipper and Douglas, 2012). These studies consistently found the negative effect of ability-focused praise. Considering the practical implications for schools, it would be important to use both types of feedback in the present study.

As a behavioral indication of intrinsic motivation, we assessed attentional engagement based on a free-choice paradigm (see Deci, 1971; Corpus and Lepper, 2007; Wiechman and Gurland, 2009) in which participants were given free time, materials, and a target task; engagement in the target task during free time was measured as intrinsic motivation. We measured visual attentional engagement in a free-choice trial using an eye-tracker, since visual attention reflects motivation or goals (e.g., Hayhoe and Rothkopf, 2011; Henderson, 2017), is highly selective, and happens early during processing (Isaacowitz, 2006); therefore, visual attention could be a more sensitive indicator of motivation than self-reported measures. Considering that some studies showed that ability-focused praise affected the praisee’s behavioral engagement (Corpus and Lepper, 2007) and visual attention (Zentall and Morris, 2012; Morris and Zentall, 2014), we predicted that ability-focused praise would negatively affect the praiser’s attentional engagement as with the effect on self-reported task enjoyment.

We conducted two identical experiments except for the performance feedback phrase. We integrated the results of the two experiments using a meta-analysis based on Cumming (2012, 2014). In both experiments, a task-related exchange and the praise-offering manipulation were conducted. Subsequently, participants worked on a difficult task, and intrinsic motivation was measured using a self-reported scale and behavioral indication. We had the same hypothesis for both the self-reported scale (task enjoyment) and the behavioral indication (attentional engagement). We hypothesized that those offering ability-focused praise would be less intrinsically motivated; specifically, they would report lower task enjoyment and would show a lower level of attentional engagement than participants in the control group.

## Method

### Participants

Participants were recruited during an introductory level university psychology class. Participants provided written informed consent. We recruited a similar sample size as in previous praise research using eye-tracking measures (Zentall and Morris, 2012; Morris and Zentall, 2014). In Experiment 1, 39 undergraduates (M_age_ = 19.19 years; SD_age_ = 0.83; 20 females) were randomly assigned to two groups (control group, *n* = 20; ability praise group, *n* = 19). In Experiment 2, 51 undergraduates (M_age_ = 19.51 years; SD_age_ = 1.46; 35 females) were randomly assigned to these groups (control group, *n* = 26; ability praise group, *n* = 25). Two female confederates (Experiment 1) and one male and one female confederates (Experiment 2) assisted the experimenter by serving as participants’ partners. The experiment was ostensibly explained as a study of an “interaction among friends and motivation.” We debriefed participants afterward. The present study was approved by the local ethics committee.

### Procedure and Materials

**FIGURE 1:**
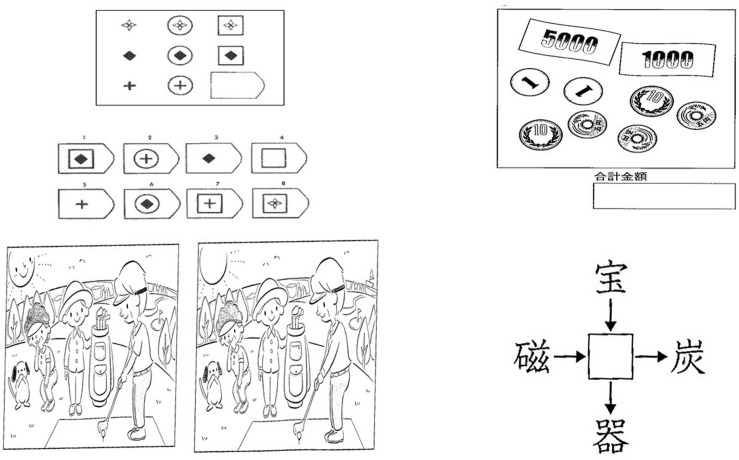
The tasks used in measuring attentional engagement. The presented tasks were a progressive matrix (upper left), a money-counting problem (upper right), a spot-the-difference task (lower left), and a kanji problem (lower right).

An overview of the procedure is shown in Table 1. First, as an icebreaker, participants and a confederate were introduced and interacted with each other. Second, a task-related exchange and the praise-offering manipulation were conducted. Participants and the confederate were asked to work on tasks separately and then to check each other’s answers and provide each other with performance feedback. Then, participants in the ability praise group were asked to offer ability-focused praise to the confederate, whereas participants in the control group were not asked to provide any additional comments. The task-related exchange and praise were conducted twice to strengthen the manipulation of the offered praise. Then, the confederate left the experimental room, ostensibly to work in another room. Third, participants worked on a difficult task and experienced failure. Fourth, participants’ task enjoyment was measured using a self-reported scale. Finally, attentional engagement was measured based on the free-choice trial. The target task and three non-target tasks were simultaneously displayed on the monitor for 10 s (Figure 1). Participants were asked to choose one of the four possible tasks and mentally work on it within 10 s. A fixation cross was presented for 3 s between each of the three blocks. Their visual attentional engagement in the task was measured through eye-tracking (50 in., Detect Corporation, Japan). This “choice and work” process was conducted three times, and the problems in each task were different every time.

**TABLE 1:**
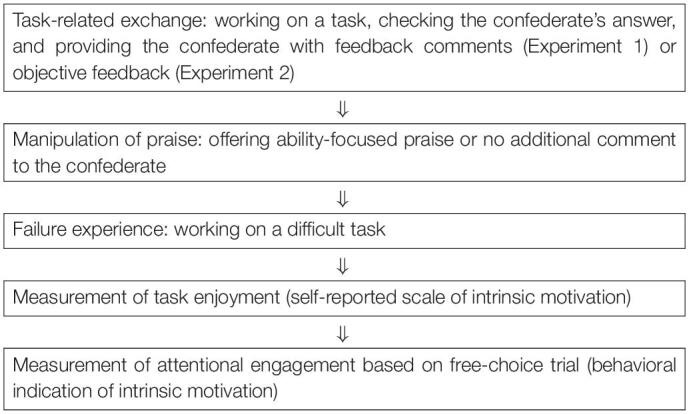
The procedure of the present experiments.

#### Performance Feedback and Manipulation of Praise^1^

In the task-related exchange, Experiment 1 used feedback comments (“You did well”) based on Mueller and Dweck (1998), and Experiment 2 used objective feedback (“Your score is Level 3”) based on Skipper and Douglas (2012). Students in the ability-focused praise group were given four ability-focused praise choices in advance (“You are smart,” “You are talented,” “You are a genius,” and “You have high potential”); they were asked to select one phrase to offer to the confederate. The confederate responded to all participants by saying “Thank you.”

#### Task Materials

Based on Mueller and Dweck (1998), Raven’s Progressive Matrices (Raven, 1976) were used as the task for participants and a confederate. In this task, a series of diagrams with a missing part needs to be completed by selecting the correct part from several options (Raven, 2000). In the task-related exchange, participants and the confederate worked on sets of 10 progressive matrices of moderate difficulty (Raven, 1976) for 4 min. In the failure experience, participants worked on a more difficult set of five progressive matrices for 2 min. For the difficult task, all participants’ scores were three points or fewer because we specifically designed the time limits and difficulty level of the task. The written instruction indicated that their scores were in the low range.

As non-target tasks displayed with the target task (Raven’s matrices), we selected a money-counting problem, a spot-the-difference problem, and a kanji problem. In the money-counting problem, the respondent had to establish the total value of coins and bills in an image. In the spot-the-difference problem, the respondent had to spot the difference between two images. In the kanji problem, the participant was shown four kanji (Chinese writing characters) and had to find a common kanji to make four phrases. The reason for this selection was that these tasks share similarities with the progressive matrices task, as they can be mentally worked on and require a similar amount of time to solve a problem.

### Measures

#### Task Enjoyment

Three items from the Intrinsic Motivation Inventory (Ryan, 1982) were used as the self-reported measure of intrinsic motivation (e.g., “I enjoyed doing this task”; Cronbach’s α = 0.72 in Experiment 1, α = 0.69 in Experiment 2). In Experiment 1, a 5-point Likert-type scale ranging from 1 (*strongly disagree*) to 5 (*strongly agree*) was used to make it easier for participants to answer the question items. Experiment 2 used a 7-point Likert-scale ranging from 1 (*strongly disagree*) to 7 (*strongly agree*) to use the same scale as in previous studies.

#### Attentional Engagement in Free-Choice Trial

As indexes of attentional engagement, we measured the number of fixations (hereafter, fixation count) and the total fixation duration (hereafter, fixation duration) in the progressive matrices task. In the progressive matrices task, finding the correct part from several options requires switching attention between diagrams and reproducing information on the diagrams (Raven, 2000). Considering that interpreting complex information often requires a high number of fixations (Holmqvist, 2011), switching attention from one part to another of the matrices would require a higher fixation count. Considering that difficult mental calculations require a longer fixation duration (Unema and Rötting, 1990), recalling and reproducing information on the diagrams would require a longer fixation duration. Therefore, both fixation count and fixation duration would reflect the amount of effort exerted in finding a solution and should function as indexes for attentional engagement in the progressive matrices task. In the analysis, areas of interest (AOIs) were manually created around the progressive matrix of each picture. We recorded participants’ fixation count and fixation duration within AOIs. An average of three AOIs fixation count and fixation duration were calculated. Details on the eye-tracking setup are shown in the Appendix.

## Results^2^

Following the recommendations of Cumming (2012, 2014), the results of Experiments 1 and 2 were integrated using a meta-analysis.

### Preliminary Analysis

Participants’ gender and the gender match between a participant and a confederate (same or different) for any variables did not affect the results (*p*s > 0.13). Therefore, gender was not included in the final analysis.

To test the validity of indexes of attentional engagement (i.e., fixation count and fixation duration), we examined the relationship between the indexes and the self-reported scale of task enjoyment. In the analysis, we used the Exploratory Software for Confidence Intervals package to obtain the integrated Pearson’s *r* and 95% confidence interval (CI). The meta-analysis showed that there were statistically significant positive correlations between fixation count and task enjoyment (*r* = 0.326, 95% CI = [0.078, 0.574]) and fixation duration and task enjoyment (*r* = 0.306, 95% CI = [0.058, 0.554]). These correlations are similar in size to that reported in previous studies (Wiechman and Gurland, 2009, *r* = 0.38). This finding indicates that the more participants enjoyed the matrices task, the more frequently and longer they fixated on the matrices task in the free-choice trial. Therefore, the indexes of visual attentional engagement can be seen as indications of intrinsic motivation.

### Main Analysis: The Effect of Praise

Table 2 presents the descriptive statistics for task enjoyment and attentional engagement across the different groups. To estimate the effect of praise, we combined the results of the two experiments and conducted a Bayes factor meta-analysis. We used the meta.ttestBF function of the BayesFactor package (Rouder and Morey, 2011; Morey and Rouder, 2018) in the R statistical programming environment (R Core Team, 2016). meta.ttestBF function can estimate the Bayes factor^3^. The Bayes factor provided by meta.ttestBF compares a null hypothesis (the standardized effect size is 0) with an alternative hypothesis (the standardized effect size is not 0) (Rouder and Morey, 2011). We also estimated the standard effect size δ for the mean difference between the ability praise group and the control group along with error variance and the g-prior across 10,000 iterations of posterior distribution sampling. The δ values were scaled such that negative values reflected lower intrinsic motivation in the ability praise groups than in the control group. A Cauchy prior was placed on the standardized effect size δ. Posterior samples were drawn *via* independent candidate Metropolis-Hastings.

**TABLE 2:** Means, standard deviations, and 95% confidence intervals of the dependent measures in Experiments 1 and 2.

	M	SD	95% CI	Possible range
**Task enjoyment**				
***Experiment 1***				1–5
Control	3.18	0.83	[2.75, 3.60]	
Ability praise	3.57	0.72	[3.17, 3.96]	
***Experiment 2***				1–7
Control	4.90	1.19	[4.29, 5.51]	
Ability praise	5.33	0.82	[4.91, 5.76]	

**Attentional engagement**				
**Fixation count**				
***Experiment 1***				
Control	7.69	6.95	[4.11, 11.26]	
Ability praise	2.78	3.92	[0.61, 4.95]	
***Experiment 2***				
Control	7.25	6.79	[3.76, 10.75]	
Ability praise	5.06	4.71	[2.64, 7.48]	
**Fixation duration (ms)**				0–10,000
***Experiment 1***				
Control	2,400	2,135	[1,302, 3,498]	
Ability praise	828	1,371	[68, 1,587]	
***Experiment 2***				
Control	2,303	2,347	[1,097, 3,510]	
Ability praise	1,645	1,716	[762, 2,527]	

*M = mean; SD = standard deviation; CI = confidence interval.*

The Bayes factor for task enjoyment suggested weak evidence for the present hypothesis (BF = 0.10). The δ was 0.398, and the 95% Bayesian credible interval was between −0.064 and 0.875, which contained zero. It did not support the hypothesis. However, there was positive evidence for the attentional engagement hypothesis (BF = 5.81 for fixation count, BF = 4.41 for fixation duration). As for the fixation count, the δ was −0.525, and the 95% Bayesian credible interval was between −1.015 and −0.052, which did not contain zero. As for fixation duration, the δ was −0.491, and the 95% Bayesian credible interval was between −0.978 and −0.026, which did not contain zero. These findings showed that attentional engagement in the ability-focused praise group was lower than that in the control group. This supported the hypothesis and indicated that the data were 5.81 times (fixation count) and 4.41 times (fixation duration) more likely to occur under our hypothesis than the null hypothesis^4^.

### Additional Analysis

We confirmed whether there were differences between the control group and the ability-focused praise group in the fixation count and fixation duration in the non-target tasks (i.e., a money-counting problem, a spot-the-difference task, and a kanji problem) and the sum of all four tasks. We conducted a Bayes factor meta-analysis similarly to the main analysis. The results of the fixation count showed that the Bayes factors for these valuables were lower than 0.88. All 95% Bayesian credible intervals of δ contained zero (for the money-counting problem, δ = −0.036, 95% CI = [−0.485, 0.406]; for the spot-the-difference task, δ = 0.157, 95% CI = [−0.290, 0.618]; for the kanji problem, δ = 0.191, 95% CI = [−0.257, 0.650]; for total time, δ = −0.270, 95% CI = [−0.735, 0.177]). The results concerning the fixation duration showed that the Bayes factors for these valuables were lower than 1.36. All 95% Bayesian credible intervals of δ contained zero (for the money-counting problem, δ = −0.091, 95% CI = [−0.543, 0.349]; for the spot-the-difference task, δ = 0.206, 95% CI = [−0.247, 0.670]; for the kanji problem, δ = 0.337, 95% CI = [−0.117, 0.807]; for total time, δ = −0.170, 95% CI = [−0.626, 0.274]). This suggests that there were no differences between the control group and the ability praise group in the non-target tasks and the sum of all four tasks.

## Discussion

Although a previous study demonstrated that ability-focused praise negatively affected the praiser’s motivation, the research contained some procedural and measurement gaps; specifically, it did not involve a face-to-face setting or measure behavioral aspects of intrinsic motivation. We conducted two experiments, asking participants to praise a confederate face-to-face and measuring the attentional engagement as a behavioral indication of intrinsic motivation. The results of task enjoyment showed that there was no significant difference between the ability-focused praise group and the control group, whereas the results of attentional engagement showed that the fixation count and fixation duration in the ability-focused praise group were significantly lower and shorter than those in the control group. We found that ability-focused praise in face-to-face setting affected only the intrinsic motivation measured as a behavioral indication.

As for attentional engagement, the results suggest a negative effect of ability-focused praise on the praiser’s attentional engagement. This finding replicates the results of Kakinuma et al. (2020) and is also consistent with research on praisees (e.g., Mueller and Dweck, 1998). This result suggests that if a student offers ability-focused praise to colleagues, it may backfire for the praiser afterward; the praiser’s motivation will decrease when they face failure. The present study extends the findings of the effect on the praiser to a real-life interaction. Furthermore, by using the behavioral measure, the present study ecologically validated the effect.

Nevertheless, there may be an alternative explanation concerning the decrease in attentional engagement in the ability-focused praise group. For example, a lower fixation count and a shorter fixation duration could reflect the process of choosing other tasks or being tired. In other words, participants in the ability praise group may have been motivated to perform different tasks, and thus their attentional engagement related to the matrices was relatively low or they may have been tired and less engaged during the free-choice trial. However, there were no differences between the control group and the ability praise group on the non-target tasks (i.e., a money-counting problem, a spot-the-difference task, and a kanji problem) and the sum of all four tasks. Therefore, the decrease in attentional engagement on a matrix task could be interpreted as a decrease in the praisers’ intrinsic motivation.

Although the current study established the effect using the behavioral measure, there was no significant effect on task enjoyment measured by the self-reported scale. This inconsistency between the results of the self-reported and behavioral measures has been found and explained in studies on motivation (Deci et al., 1999; Wiechman and Gurland, 2009). The explanations may be related to the difference between the present result and previous research, which found the effect on self-reported task enjoyment (Kakinuma et al., 2020). In the present study, participants worked on a task with the confederate and offered the praise in person, whereas, in the study by Kakinuma et al. (2020), participants observed a person doing a task through a monitor and wrote sentences of praise on a paper in another room. In other words, the participants in the present study were more exposed to the confederate and might have been more concerned or worried about the impression they made on the confederate. The studies on motivation suggested that the self-reported measure of intrinsic motivation is more likely to be interfered with by an intentional process than the behavioral measure (Deci et al., 1999; Wiechman and Gurland, 2009). Furthermore, research on implicit measures has shown that when participants are asked to consider additional information after an experimental manipulation, the experimental effects are apparent only for implicit measures and not for explicit measures (Gawronski and LeBel, 2008). In the present study, the intentional process, that is, participants’ concern about the impression they produced on others, may have weakened the effect on the self-reported task enjoyment. Behavioral measures may tend to be free from interference by other factors and may be more sensitive to an experimental effect than self-reported measures.

The present study has practical implications for social and educational contexts. Recently, praise feedback has been incorporated into various educational policies. For example, the Japanese government’s course guidelines argue that teachers need to provide positive feedback to their students (Japanese Ministry of Education, Culture, Sports, Science and Technology, 2018). Some schools encourage students to praise each other (e.g., Fukuoka-Ken Board of Education, 2015). Whereas previous findings on feedback have supported these educational policies (e.g., Topping, 1998; Cho and Cho, 2011; Deiglmayr, 2018), the present study provides reasons to approach these policies with caution. The present findings suggest that ability-focused praise has a negative effect on the giver. Nevertheless, we could not strongly insist that people should avoid offering ability-focused words because the present study did not include people offering differently oriented praise as a control group.

Limitations of the present study should be noted and used to suggest the direction of future research. First, the mean differences for fixation count and fixation duration in Experiment 2 were small compared with Experiment 1. One possible reason is that the performance feedback phrase used in Experiment 2 (“Your score is Level 3”) sounded matter-of-fact or impersonal. Then, ability-focused praise following this phrase would be less emotionally laden, and participants’ engagement in offering ability-focused praise would be, therefore, lower in Experiment 2. Research on the “saying is believing” effect showed the importance of commitment or satisfaction in making statements (Briñol et al., 2012), and research on praise emphasizes the importance of flexibility in offering praise (Henderlong and Lepper, 2002). To make praise more engaged and flexible, future research should ask participants to generate praise words themselves, instead of using only words that the experimenter provides. As a second limitation, the present study used the mere exchange of feedback message. Peer feedback studies proposed a two-way, multi-turn dialogue as a method of peer feedback learning (Deiglmayr, 2018). Future research should incorporate the perspective of interactive dialogue into the investigation of the effects of praise and examine whether a praisee’s response or behavior may moderate the effect on the praiser.

## Data Availability Statement

The raw data supporting the conclusions of this article will be made available by the authors, without undue reservation.

## Ethics Statement

The studies involving human participants were reviewed and approved by the Doshisha University Faculty of Psychology Research Ethics Committee. The patients/participants provided their written informed consent to participate in this study.

## Author Contributions

KK, FN, KS, and HT collected the data. KK performed the statistical analysis. AT assisted in the statistical analysis and contributed to supervision. KK drafted the manuscript. KK and AT contributed to manuscript revision. All authors contributed to the conception and design of the study. All authors contributed to the article and approved the submitted version.

## Conflict of Interest

The authors declare that the research was conducted in the absence of any commercial or financial relationships that could be construed as a potential conflict of interest.

## Footnotes

1 To make the situation seem as real and natural as possible, participants in both groups and a confederate were asked to provide performance feedback corresponding to their partner’s score. In the ability praise group, participants were asked to offer ability-focused praise only if the confederate earned eight points or more on the task; in order to ensure feedback, we asked the confederate in advance to obtain eight points. In addition, in order to inhibit participants from comparing their score with the confederate’s score, we asked the confederate to check participants’ answers, ensuring that participants also earned eight points.

2 When analyzing attentional engagement, 5 participants (Experiment 1) and 11 participants (Experiment 2) were eliminated because of a technical measurement problem. There was no significant difference in task enjoyment between the excluded participants and the included participants (*p*s > 0.08).

3 The Bayes factor is a ratio that contrasts the likelihood of the data fitting under the null hypothesis with the likelihood of fitting under the alternative hypothesis (Jarosz and Wiley, 2014). As the Bayes factor increases, there is more evidence in support of the alternative hypothesis and less in favor of the null hypothesis. For example, Bayes factor = 3 suggests that the data are three times more likely to occur under the alternative hypothesis than the null hypothesis. The guideline for interpretation of Bayes factors (Raftery, 1995) states that Bayes factors between 1 and 3 represent weak support for an alternative hypothesis compared with a null hypothesis, Bayes factors between 3 and 20 represent positive support, and Bayes factors between 20 and 150 represent strong support. The Bayes factor is computed *via* Gaussian quadrature.

4 Additionally, *t*-tests were conducted to compare the variables (task enjoyment, fixation count, and fixation duration) of the control group with the variables of the ability-focused praise group for each experiment. The statistics of the comparisons were as follows: task enjoyment (*t*(30) = 1.416, *p* = 0.167 for Experiment 1; *t*(32) = 1.232, *p* = 0.227 for Experiment 2), fixation count (*t*(30) = 2.414, *p* = 0.022 for Experiment 1; *t*(32) = 1.096, *p* = 0.281 for Experiment 2), and fixation duration (*t*(30) = 2.440, *p* = 0.021 for Experiment 1; *t*(32) = 0.934, *p* = 0.357 for Experiment 2). Although the mean differences of fixation count and duration were not statistically significant in Experiment 2, the sampling variability of *p* values tends to be larger (Cumming, 2012); therefore, we conducted a meta-analysis and combined the results of the two experiments. Recently, it has been recommended to emphasize the combination of multiple results of similar experiments, rather than simply focusing on whether or not there is an effect within a single experiment. The accumulation of evidence, by meta-analyzing even two studies, can usefully increase precision (Cumming, 2012). We should note that the two experiments would be homogeneous. We calculated *Q* values, which are the measure of heterogeneity or variability between study means. A *Q* value notably greater than (*k* − 1) indicates that the studies are heterogeneous (*k* is the number of studies in the meta-analysis), whereas a *Q* value smaller than or close to (*k* − 1) indicates that the studies are homogeneous (Cumming, 2012). The results showed that *Q* values of the variables were smaller or close to *df* (2 − 1 = 1); task enjoyment (*Q* = 0.017, *p* = 0.896), fixation count (*Q* = 0.890, *p* = 0.345), fixation duration (*Q* = 1.146, *p* = 0.284). These results suggested that the study-to-study variation in the means could be explained by sampling variation. Therefore, we provided the results of meta-analysis as main evidence for the hypotheses.

## Appendix

Details of the eye-tracking setup. Participants took a seat 140 cm distance from the screen (1,920 × 1,080 pixels and physical size of 110 × 60 cm). We recorded eye movements using the eye-tracking QG-Plus software (Eyetech Digital System Corporation) with a monocular sampling rate of 50 Hz and performed a 9-point calibration. We accepted calibrations with score less than 30. In data analysis, we drew an area of interest (AOI) with a 33 × 20 cm rectangle around the progressive matrix.
